# The complete mitochondrial genome of common Shelduck Shengjin Lake *Tadorna tadorna*

**DOI:** 10.1080/23802359.2019.1667900

**Published:** 2019-09-19

**Authors:** Gang Liu, Qingyue Li, Zhizong Gong

**Affiliations:** School of Life Sciences, Anhui Medical University, Hefei, P. R. China

**Keywords:** *Tadorna tadorna*, Shengjin Lake, complete mitochondrial genome sequences

## Abstract

The complete mtDNA of Shengjin Lake *Tadorna tadorna* 16622 bp in length, containing 13 protein-coding genes, 2 rRNAs, 22 tRNAs and one D-loop. Mitochondrial genes of the Shengjin Lake *Tadorna tadorna* was arranged in the same order and orientation as those of reported *Tadorna tadorna*. All the start codon of the protein-coding genes is ATG, TAA is the most frequent stop codon. The 12S rRNA and 16S rRNA genes are located between the tRNA^Leu^ and tRNA^Phe^ genes and separated by the tRNA^Val^ gene. The control region, located between the tRNA^Glu^ and tRNA^Phe^ genes and is rich in A and T. Phylogenetic trees show Shengjin Lake *Tadorna tadorna* has close relative with *Cairina moschata.*

Shengjin Lake is the most important lakes in the middle and lower Yangtze River floodplain. It is important stopover and wintering grounds for many East Asian-Australasian migratory geese (Barter et al. 2005; Cao et al. 2011). The seasonally inundated wetlands support an abundance of waterbird populations, including global Anseriformes including common shelduck *Tadorna tadorna* (Barter et al. 2005; Cao et al. 2011). The common shelduck is a long-distance migratory waterfowl and an important wetland indicator species within the family Anatidae and order Anseriformes, breeds throughout the temperate and subtropical Asia and Eurasia, which winter in the middle and lower reaches of Yangtze River of China. This duck belongs to the family Anatinae of the waterfowl family Anatidae.

In the present study, we sequenced the complete mtDNA of *Tadorna tadorna* winting at Shengjin Lake. The feather samples were collected from *Tadorna tadorna* rescued in Shengjin Lake National Nature Reserve (E117°0′42.55″, N30°20′30.73″), Anhui Province of China in October 2017. The samples were stored at −20 °C in the Cancer Cell Biology Laboratory, School of Life Sciences, Anhui Medical University (Sample code is AHMU-WB20171018). The complete mitochondrial genome has been submitted to GenBank (accession number MN258348). The complete mtDNA of Shengjin Lake *Tadorna tadorna* is 16622 bp in length, containing 13 protein-coding genes, 2 rRNAs, 22 tRNAs and one D-loop. Mitochondrial genes of the Shengjin Lake *Tadorna tadorna* was arranged in the same order and orientation of reported *Tadorna tadorna* (GenBank accession number) (Sun et al. 2017). Comparing to KU140668, there is 148 bp variable in the new mtDNA (Sun et al. 2017). All the mitogenome genes were encoded on the H strand except for one protein-coding gene (ND6) and eight tRNA genes, similar to typical Anseriformes mtDNAs (Liu et al. 2013). The composition of base composition in Shengjin Lake *Tadorna tadorna* indicating a remarkably high AT content and a low GC content, which is similar observed in other Anatidae mt genomes (Liu et al. 2013, 2014). All the start codon of the protein-coding genes is ATG, and TAA is the most frequent stop codon. The lengths of the 12S rRNA and 16S rRNA genes are 991 bp and 1604 bp, respectively, and are located between the tRNA^Leu^ and tRNA^Phe^ genes and separated by the tRNA^Val^ gene. The control region, located between the tRNA^Glu^ and tRNA^Phe^ genes is 1061 bp and is rich in A and T.

Phylogenetic trees were estimated using ML and BI methods, based on the complete mtDNA of seven ducks, and corresponding *Gallus gallus* (NC_001323) sequence was used as an outgroup, sharing similar topologies and high node support values (Figure 1). Shengjin Lake *Tadorna tadorna* has close relative with *Cairina moschata.*

**Figure 1.**
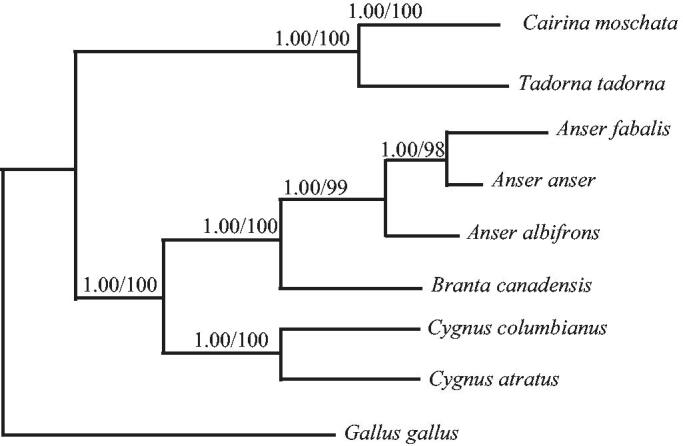
Phylogenetic trees based on complete mtDNA sequeces of seven ducks utilized the ML and BI methods. Numbers at each node are bootstrap values from three analyses (maximum likelihood/Bayesian inference). Notes: *Cairina moschata*: NC_010965, *Tadorna Tadorna*, *Anser albifrons*: NC_004539, *Anas formosa*: NC_015482, *Anser anser*: NC_011196, *Branta canadensis*: NC_007011, *Cygnus columbianus:* NC_007691, *Cygnus atratus*: NC_012843.

## Nucleotide sequence accession number

The complete mtDNA sequence of Shengjin Lake *Tadorna tadorna* has been assigned with GenBank accession number MN258348.
